# Rare fungal bloodstream infections in pediatric patients: a case series

**DOI:** 10.1590/S1678-9946202668034

**Published:** 2026-05-15

**Authors:** Nicole Feitosa Ximenes Ferreira, Giovana Fernandes Zorzan, André Ricardo Araújo da Silva

**Affiliations:** 1 Universidade Federal Fluminense, Curso de Medicina, Niterói, Rio de Janeiro, Brazil; 2 Universidade Federal Fluminense, Departamento Materno-Infantil, Niterói, Rio de Janeiro, Brazil; 3 Grupo Prontobaby, Comissão de Controle de Infecção Hospitalar, Rio de Janeiro, Rio de Janeiro, Brazil

**Keywords:** Rare fungi, Invasive fungal infections, Pediatric patients, Fungemia, Hospitalization

## Abstract

Fungemia caused by rare fungal species has been increasingly recognized in both immunocompromised and immunocompetent children. Our aim is to characterize the clinical and microbiological features of rare fungal bloodstream infections in hospitalized pediatric patients. A retrospective, descriptive study was conducted including all cases of fungemia due to rare yeasts in patients admitted to two tertiary pediatric hospitals between 2021 and 2025. Fungal species identification, anatomical sites of infection, antifungal therapeutic regimens, and clinical outcomes were systematically analyzed. In total, 13 cases of rare fungal bloodstream infections were identified among 13 patients from 2,555 blood cultures obtained during the study period, representing an incidence of 0.5%. The predominant species isolated were *Saccharomyces cerevisiae* (n=5), *Wickerhamomyces anomalus* (n=3), *Candid orthopsilosis* (n=2), *Yarrowia lipolytica* (n=1), *Meyerozyma guilliermondii* (n=1), and *Trichosporon asahii* (n=1). The median age at infection was 31 months (range: 0–178 months), with 92.3% (12/13) of patients presenting underlying medical conditions. The median interval from hospital admission to fungemia onset was 19 days (range: 0–73 days). Amphotericin B deoxycholate was the most frequently employed initial antifungal agent (n=5), followed by liposomal amphotericin B (n=4). Modification of antifungal therapy was required in five cases (38.5%). Microbiological clearance was achieved in all patients on subsequent blood cultures. The 30-day all-cause mortality rate was 15.4% (2/13). We concluded that rare fungal bloodstream infections occurred predominantly in patients with underlying comorbidities and prolonged hospital stays; nevertheless, the majority showed favorable clinical responses with appropriate antifungal therapy and achieved fungal eradication from the bloodstream.

## INTRODUCTION

Invasive fungal infections caused by rare or emerging fungal pathogens have received increasing clinical and epidemiological attention in recent decades, largely attributable to the expanding population of immunocompromised and critically ill patients^1^. In immunocompetent hosts, these fungal species typically cause benign, self-limited infections confined to cutaneous or mucosal surfaces, such as dermatophytoses or superficial mycoses^2^. However, in immunosuppressed individuals—particularly pediatric patients whose immune systems are developmentally immature—these organisms may precipitate invasive and disseminated infections characterized by rapid progression and severe clinical manifestations^2^. Although relatively uncommon, these infections are associated with significant mortality and present substantial diagnostic and therapeutic challenges, as many causative species exhibit intrinsic or acquired resistance to conventional antifungal agents. Consequently, enhanced recognition of these emerging pathogens and refinement of diagnostic and therapeutic approaches are imperative to improve patient outcomes^2-4^.

The category of rare fungi encompasses species from diverse genera including *Fusarium, Geotrichum*, *Saprochaete, Trichosporon*, *Magnusiomyces*, and *Rhodotorula*, as well as uncommon non-*albicans Candida* species^3,4^. Several of these organisms are commensal members of the human microbiota that may transition to pathogenic behavior under conditions of immune dysfunction, emerging as causative agents of invasive disease. The considerable heterogeneity among species and their variable susceptibility patterns to different antifungal drug classes underscore the critical importance of ongoing mycological surveillance and continuous updating of diagnostic protocols^3,4^.

The escalating frequency of reported infections caused by rare fungal species reflects the increasing complexity of opportunistic mycoses within vulnerable hospital populations and immunologically compromised hosts^5^. Robust epidemiological surveillance systems, timely diagnosis, and judicious use of both antifungal agents and probiotic preparations are essential strategies to prevent nosocomial outbreaks and reduce mortality attributable to these emerging pathogens. In light of these considerations, the objective of this study was to characterize a case series of invasive infections caused by rare fungi in pediatric patients hospitalized at two tertiary care centers.

## MATERIALS AND METHODS

### Ethics

The study protocol was reviewed and approved by the Research Ethics Committee of the Fluminense Federal University School of Medicine (approval No. 7.707.721, dated July 15, 2025). Given the retrospective nature of the study, the requirement for informed consent was waived.

### Study design and setting

A retrospective, descriptive study was conducted to identify all cases of rare fungal bloodstream infections among pediatric patients admitted to two tertiary care hospitals in Rio de Janeiro, Brazil, from January 1, 2021, to June 30, 2025. Both institutions are located in Rio de Janeiro and provide exclusive pediatric care to patients with private health insurance coverage, including admissions through emergency departments, inter-hospital transfers, and home healthcare service referrals. Hospital 1 comprises 140 inpatient beds, including 45 pediatric intensive care unit (PICU) beds, while Hospital 2 maintains 45 inpatient beds, including 20 PICU beds and 16 pediatric cardiac surgical intensive care unit beds. Neither hospital has a dedicated neonatal intensive care unit (NICU); therefore, neonatal patients were admitted to the PICUs.

### Study population

Inclusion criteria comprised patients aged 0 to <18 years with laboratory-confirmed isolation of rare fungal species from blood cultures obtained by attending physicians based on clinical suspicion of bloodstream infection. Patients transferred to other healthcare facilities within the first 24 h of admission were excluded from the analysis.

### Definitions

For the purposes of this article, rare fungi were defined following the classification established by Arendrup *et al*.^3^, encompassing non-*albicans Candida* species with the exception of *Nakaseomyces glabrata, Candida tropicalis, Candida parapsilosis*, and *Pichia kudriavzevii*, which are considered common pathogens.

For epidemiological classification, isolation of a rare fungal species from blood culture in the presence of clinical signs and symptoms consistent with a bloodstream infection, as defined by the Brazilian Health Regulatory Agency^6^, was classified as an acute infection episode. If two or more isolates were recovered from blood samples within the same episode, only one was included in the analysis. Recovery of the same fungal species from cultures obtained within 7 days of the initial positive culture was categorized as persistent infection, whereas isolation of the same or a different rare fungal species occurring ≥14 days after the index episode was designated as a new infection episode.

### Infection control aspects

Both hospitals have maintained a continuous infection control team since 2000, with well-established measures to prevent healthcare-associated infections, including biannual air quality analysis of critical care units.

### Microbiological methods

All blood culture specimens were processed using an automated continuous monitoring system (BD BACTEC™ Peds Plus/F culture vials; BD BACTEC™ 9240, Becton Dickinson, Franklin Lakes, NJ, USA). The BD BACTEC™ Mycosis IC/F Culture Vials were not available during the study period. Fungal isolates that underwent species-level identification were sent to a partner laboratory, and antifungal susceptibility testing was performed following the Clinical & Laboratory Standards Institute (CLSI) guidelines to determine minimum inhibitory concentrations (MICs)^7^, resistance patterns, and clinical breakpoints for each isolated organism. An automated system was used to determine the MIC (VITEK 2^®^ Yeast Panel), alongside broth microdilution to confirm the susceptibility of fungal isolates to antifungal agents, and CHROMagar for the identification of mixed colonies

### Data collection and statistical analysis

Clinical and demographic data were extracted from electronic medical records and presented using descriptive statistics. Analyzed variables included primary reasons for hospitalization, underlying comorbidities, antifungal therapeutic regimens administered following fungal detection, and 30-day clinical outcomes. Categorical variables were compared using the chi-square test or Fisher's exact test when appropriate, while continuous variables were analyzed using the Mann-Whitney U test. A two-tailed p-value <0.05 was considered statistically significant. All statistical analyses were performed using SPSS statistical software (version 16.0, IBM, Armonk, NY, USA).

## RESULTS

### Epidemiological and microbiological characteristics

During the 4.5-year study period (January 1, 2021, to June 30, 2025), a total of 2,555 blood cultures were obtained, of which 13 cases of rare fungal bloodstream infections were identified in 13 distinct patients, representing an overall incidence of 0.5%. The distribution of causative organisms was as follows: *Saccharomyces cerevisiae* (n=5, 31.3%), *Wickerhamomyces anomalus* (n=3, 18.8%), *Candida orthopsilosis* (n=2, 15.4%), and single isolates of *Yarrowia lipolytica, Meyerozyma guilliermondii* and *Trichosporon asahii* (n=1 each, 7.7%).

### Patient demographics and clinical characteristics

The median age at infection diagnosis was 31 months (range: 0–178 months, IQR=93.5). The majority of patients (92.3%, 12/13) presented with at least one underlying medical condition. The median interval from hospital admission to documented fungemia was 19 days (range: 0–73 days, IQR=28.5), indicating a predominance of healthcare-associated infections. All children had a central venous catheter *in situ* prior to the bloodstream infection, with a median duration of 19.5 days (IQR=11). In total, four patients received parenteral nutrition before the infection and during their current hospital stay, seven children (53.8%) underwent surgical procedures, and none received probiotics prior to laboratory-confirmed isolation from blood cultures.

### Antifungal treatment and clinical outcomes

Amphotericin B deoxycholate was the most frequently prescribed initial antifungal agent (n=5, 38.5%), followed by liposomal amphotericin B (n=4, 30.8%). Modification of the initial antifungal regimen was required in four cases (30.8%) due to a lack of clinical response. Microbiological clearance, defined as negative follow-up blood cultures, was achieved in all 13 patients (100%). The 30-day all-cause mortality rate was 15.4% (2/13 patients). Table 1 summarizes detailed clinical characteristics, reasons for blood collection, underlying conditions, therapeutic interventions, and outcomes for individual patients.

**Table 1:** Clinical characteristics, underlying conditions, antifungal therapy, and outcomes of pediatric patients with rare fungal bloodstream infections (Rio de Janeiro, Brazil, 2021–2025)

Case	Year of occurrence	Isolated agent	Site of isolation	Underlying condition / Previous antifungal or antibiotic treatment	Reason for blood collection	Treatment	Fungal clearance within 7 days	Clinical improvement after 7 days	30-day outcome
1	2021	*Candida orthopsilosis*	Blood	Dialysis-dependent renal failure/No previous antifungal or antibiotic treatment	Clinical worsening, fever	Lipid complex amphotericin B (15 days)	Yes	Yes	Discharged
2	2022	*Meyerozyma guilliermondii*	Catheter blood	Non-Hodgkin lymphoma + hemophagocytic syndrome /Previous antibiotic treatment (3 days)	Fever, clinical typhlitis	Liposomal amphotericin B (8 days) and caspofungin (6 days)	Yes	No. Required antifungal treatment modification	Discharged
3	2023	*Saccharomyces cerevisiae*	Blood	Central nervous system tumor/ Previous antibiotic treatment	Leukopenia, thrombocytopenia, fever, elevated CRP	Amphotericin B deoxycholate (11 days) and Voriconazole (21 days)	Yes	No. Required antifungal treatment modification	Still hospitalized
4	2023	*Saccharomyces cerevisiae*	Catheter blood	Malnutrition / Tuberculosis. /Previous antibiotic treatment (38 days)	Persistent fever	No antifungal therapy – catheter removed	Yes	Yes	Discharged
5	2023	*Wickerhamomyces anomalus*	Blood	Hydrocephalus / prematurity / non-progressive chronic encephalopathy/ Previous antibiotic treatment (14 days)	Clinical worsening, persistent fever	Amphotericin B deoxycholate (3 days) and caspofungin (14 days)	Yes	No. Required antifungal treatment modification	Discharged
6	2023	*Saccharomyces cerevisiae*	Blood	Congenital heart disease / Previous antibiotic treatment (25 days)	Clinical sepsis	Amphotericin B deoxycholate (14 days)	Yes	Yes	Still hospitalized
7	2023	*Wickerhamomyces anomalus*	Blood	Congenital heart disease / Previous antibiotic treatment (9 days)	Leukopenia, thrombocytopenia, fever, elevated CRP	Fluconazole (6 days) and liposomal amphotericin B (14 days)	Yes	No. Required antifungal treatment modification	Discharged
8	2023	*Wickerhamomyces anomalus*	Blood	Congenital heart disease / prematurity / Previous antibiotic treatment (15 days) and antifungal (6 days)	Clinical worsening, persistent fever	Liposomal amphotericin B (21 days)	Yes	Yes	Death
9	2023	*Candida orthopsilosis*	Blood	Necrotizing pneumonia / Previous antibiotic treatment (1 day)	Persistent fever	Amphotericin B deoxycholate (21 days)	Yes	Yes	Discharged
10	2023	*Yarrowia lipolytica*	Blood	Primary immunodeficiency / previous antibiotic treatment (56 days) and antifungal therapy (5 days)	Persistent fever	Amphotericin B deoxycholate (14 days)	Yes	Yes	Still hospitalized
11	2024	*Saccharomyces cerevisiae*	Blood	Pneumonia/ prematurity / Previous antibiotic treatment (9 days)	Persistent fever	Liposomal amphotericin B (10 days)	Yes	Yes	Discharged
12	2024	*Saccharomyces cerevisiae*	Blood	Necrotizing pneumonia / Previous antibiotic treatment (16 days)	Persistent fever	Micafungin (21 days)	Yes	Yes	Discharged
13	2025	*Trichosporon asahii*	Blood	Acute lymphoblastic leukemia	Febrile neutropenia	Voriconazole (8 days) and liposomal amphotericin B (18 days)	Yes	No. Required antifungal treatment modification	Death

### Antifungal therapy duration

The duration of antifungal therapy following the isolation of the causative organism ranged from 10 to 26 days, with a median treatment duration of 17 days. Notably, antifungal therapy was not initiated in one case due to central venous catheter removal.

### Antifungal susceptibility profile

*In vitro* antifungal susceptibility testing was performed on isolates from five patients (38.5%). Table 2 shows the susceptibility profile and the minimum inhibitory concentration (MIC) of the tested antifungal agents.

**Table 2:** Antifungal susceptibility profiles and minimum inhibitory concentrations (MICs) of rare fungal isolates from pediatric bloodstream infections (Rio de Janeiro, Brazil, 2021–2025)

Case	Isolated agent	Amphotericin B	Caspofungin	Fluconazole	Micafungin	Voriconazole
2	*Meyerozyma guilliermondii*	NT	0.25*	NT**	0.5	NT
5	*Wickerhamomyces anomalus*	0.5	0.25	4	<0.06	<0.1
7	*Wickerhamomyces anomalus*	0.5	<0.12	4	<0.06	0.3
8	*Wickerhamomyces anomalus*	0.5	0.25	4	<0.06	0.5
9	*Candida orthopsilosis*	0.5	0.5	<0.5	0.5	<0.1
10	*Yarrowia lipolytica*	NT	0.5	2	0.5	<0.1

* Values expressed in mg/L from VITEK 2^®^;

** not tested.

### Overall fungal bloodstream infection burden

Among the 2,555 blood cultures obtained during the study period, fungi (including both rare and common species) were isolated in 154 instances, accounting for 6.0% of all positive cultures. Rare fungal species represented 8.4% (13/154) of all fungal isolates and 0.5% (13/2,555) of total positive blood cultures, underscoring the infrequent occurrence of these pathogens.

## DISCUSSION

Although uncommon, fungemia in pediatric populations can result in substantial morbidity and adverse clinical outcomes. The incidence of candidemia in pediatric intensive care units may reach 5 per 1,000 admissions, with associated mortality rates approaching 20%^8,9^. When examining the overall burden of bloodstream infections in hospitalized patients, fungi have been reported to account for 5% to 7.8% of all cases^10,11^. Our findings, with fungi representing 6.0% of all positive blood cultures, are consistent with these previously published data. However, when restricting the analysis to rare fungal species, these organisms accounted for only 0.5% of all positive cultures, confirming the relatively uncommon nature of these infections.

In recent years, an increasing number of case reports and nosocomial outbreaks involving invasive infections caused by rare and emerging fungal pathogens, including cases of fungemia, have been documented in the literature^12-15^. These reports highlight the evolving epidemiology of invasive mycoses in vulnerable patient populations.

In our cohort, nearly all patients (92.3%) presented with underlying comorbidities and experienced prolonged hospitalizations exceeding 15 days (median: 19 days). This observation aligns with previous reports in which hematologic malignancies and primary or acquired immunodeficiency states have been identified as major risk factors for rare fungal infections^16-18^. The extended duration of hospitalization likely reflects both the severity of the underlying conditions and the healthcare-associated nature of these infections, with increased exposure to invasive medical devices and broad-spectrum antimicrobial therapy.

Throughout the study period, no temporal clustering of cases or environmental contamination was identified that would suggest a common source outbreak. This finding contrasts with numerous reports in the literature describing nosocomial outbreaks of rare fungal infections, frequently originating from specific hospital sectors or localized units such as pediatric intensive care units^19,20^. The sporadic distribution of cases in our study suggests diverse acquisition pathways and underscores the importance of maintaining broad surveillance for these emerging pathogens.

Of particular clinical significance is *Candida pelliculosa*, taxonomically reclassified as *Wickerhamomyces anomalus*, which has been characterized as an opportunistic pathogen responsible for infections in immunocompromised hosts, as we found in three patients. This species has demonstrated notable epidemiological importance through its association with healthcare-associated candidemia outbreaks, particularly affecting hospitalized neonates. The clinical relevance of this organism was exemplified by the first documented outbreak of *W. anomalus* candidemia in South Korea in 2015, which involved 11 patients across intensive care units and general wards^21^.

Considering our cohort, *Saccharomyces cerevisiae* was the most common agent isolated. Additionally, a case report from South Korea described *Saccharomyces cerevisiae* fungemia in an extremely preterm neonate, with the bloodstream infection documented within 24 h of initiating probiotic supplementation with Ultra-Levure^®^ (*S. boulardii*), in the presence of a central venous catheter serving as a major predisposing risk factor^22^. The occurrence of infections by this fungus is associated with the use of probiotics, with reports of cases even in patients hospitalized in a bed adjacent to another child receiving a probiotic preparation containing *S. boulardii* for the treatment of diarrhea^23^.

The majority of patients in our series received amphotericin B-based formulations as initial therapy, with favorable clinical responses and documented microbiological clearance in subsequent blood cultures. Nevertheless, modification of the initial antifungal regimen was required in five patients (38.5%) due to an inadequate clinical response or other therapeutic considerations. Of these five patients, only one died, likely due to the underlying disease—acute lymphoblastic leukemia. Because we studied a small number of patients, a multivariate analysis was not possible to determine which factors contributed to the unfavorable outcome.

A significant challenge in managing rare fungal infections is the absence of established Clinical and Laboratory Standards Institute (CLSI) or European Committee on Antimicrobial Susceptibility Testing (EUCAST) interpretive breakpoints for most of the species identified in our study. This lack of standardized susceptibility criteria complicates evidence-based treatment decisions for clinicians managing similar cases in other institutions. In clinical practice, therefore, therapeutic efficacy must be assessed primarily through clinical response parameters and serial blood culture negativity rather than relying solely on in vitro susceptibility data.

The 30-day all-cause mortality rate in our cohort was 15.4% (2/13 patients), substantially lower than the 65.3% mortality reported by Kaur *et al*.^24^ in a comparable pediatric population. Several factors may explain this difference. The cohort described by Kaur *et al.* ^24^ included younger patients with more rapid infection onset following hospital admission, and *Wickerhamomyces anomalus*—a species associated with particularly poor outcomes—was among the most frequently isolated organisms in that study. The relatively favorable outcomes in our series may reflect timely recognition and initiation of appropriate antifungal therapy, as well as comprehensive supportive care in tertiary pediatric centers.

### Limitations

This study has several important limitations that warrant consideration. First, data were collected from only two institutions located in the same metropolitan area, which may limit the generalizability of our findings to other geographic regions or healthcare settings. Nevertheless, our results suggest that similar cases may be occurring in other institutions but remain underdiagnosed due to insufficient clinical suspicion, limited mycological laboratory capabilities, or the unavailability of comprehensive antifungal susceptibility testing. Second, the inherent rarity of these fungal species and the paucity of well-characterized reference isolates preclude the establishment of robust clinical breakpoints, which may impede optimal therapeutic decision-making. Finally, the retrospective design of this study may have introduced selection bias and limited our ability to capture all relevant clinical variables that could influence patient outcomes.

## CONCLUSIONS

Cases of fungemia caused by rare fungi occurred predominantly in patients with previous comorbidities and prolonged hospital stays. However, most cases showed a favorable clinical course and a good response to the treatments administered, most of which were based on amphotericin B formulations. *Saccharomyces cerevisiae and Wickerhamomyces anomalus were the most frequently isolated agents.*

These findings underscore the importance of maintaining a high index of suspicion for rare fungal pathogens in immunocompromised pediatric patients with prolonged hospitalizations and emphasize the need for enhanced mycological surveillance, improved diagnostic capabilities, and further research to establish evidence-based treatment guidelines for these emerging infections.

## Data Availability

The anonymized dataset generated during this study is available from the corresponding author upon reasonable request.
